# Projection Micro-Stereolithography to Manufacture a Biocompatible Micro-Optofluidic Device for Cell Concentration Monitoring

**DOI:** 10.3390/polym15224461

**Published:** 2023-11-19

**Authors:** Lorena Saitta, Emanuela Cutuli, Giovanni Celano, Claudio Tosto, Dario Sanalitro, Francesca Guarino, Gianluca Cicala, Maide Bucolo

**Affiliations:** 1 Department of Civil Engineering and Architecture, University of Catania, Via Santa Sofia 64, 95125 Catania, Italy; giovanni.celano@unict.it (G.C.); claudio.tosto@unict.it (C.T.); gianluca.cicala@unict.it (G.C.); 2 Department of Electrical Electronic and Computer Science Engineering, University of Catania, Via Santa Sofia 64, 95125 Catania, Italy; dario.sanalitro@unict.it (D.S.); maide.bucolo@unict.it (M.B.); 3 Department of Biomedical and Biotechnological Science, University of Catania, Via Santa Sofia 89, 95123 Catania, Italy; francesca.guarino@unict.it; 4 INSTM-UDR CT, Viale Andrea Doria 6, 95125 Catania, Italy

**Keywords:** 3D printing, vat photopolymerization, photocurable biocompatible resins, micro-optics, microfluidics, two-phase flow detection, cell concentration detection

## Abstract

In this work, a 3D printed biocompatible micro-optofluidic (MoF) device for two-phase flow monitoring is presented. Both an air–water bi-phase flow and a two-phase mixture composed of micrometric cells suspended on a liquid solution were successfully controlled and monitored through its use. To manufacture the MoF device, a highly innovative microprecision 3D printing technique was used named Projection Microstereolithography (PμSL) in combination with the use of a novel 3D printable photocurable resin suitable for biological and biomedical applications. The concentration monitoring of biological fluids relies on the absorption phenomenon. More precisely, the nature of the transmission of the light strictly depends on the cell concentration: the higher the cell concentration, the lower the optical acquired signal. To achieve this, the microfluidic T-junction device was designed with two micrometric slots for the optical fibers’ insertion, needed to acquire the light signal. In fact, both the micro-optical and the microfluidic components were integrated within the developed device. To assess the suitability of the selected biocompatible transparent resin for optical detection relying on the selected working principle (absorption phenomenon), a comparison between a two-phase flow process detected inside a previously fully characterized micro-optofluidic device made of a nonbiocompatible high-performance resin (HTL resin) and the same made of the biocompatible one (BIO resin) was carried out. In this way, it was possible to highlight the main differences between the two different resin grades, which were further justified with proper chemical analysis of the used resins and their hydrophilic/hydrophobic nature via static water contact angle measurements. A wide experimental campaign was performed for the biocompatible device manufactured through the PμSL technique in different operative conditions, i.e., different concentrations of eukaryotic yeast cells of *Saccharomyces cerevisiae* (with a diameter of 5 μm) suspended on a PBS (phosphate-buffered saline) solution. The performed analyses revealed that the selected photocurable transparent biocompatible resin for the manufactured device can be used for cell concentration monitoring by using ad hoc 3D printed micro-optofluidic devices. In fact, by means of an optical detection system and using the optimized operating conditions, i.e., the optimal values of the flow rate FR=0.1 mL/min and laser input power P∈{1,3} mW, we were able to discriminate between biological fluids with different concentrations of suspended cells with a robust working ability $R^{2}=0.9874$ and $R_{adj}^{2}=0.9811$.

## 1. Introduction

In the field of life sciences and biomedical research, the precise evaluation of cellular concentrations is of paramount importance. Measuring cell concentration involves determining the number of cells in a given volume. This information is crucial for various applications, including cell culture [1,2,3], drug development [4,5], disease diagnostics [6,7], and tissue engineering [8,9]. To meet the ever-growing demands for efficient and high-throughput cell analysis, microfluidic devices have emerged as innovative tools, offering control, sensitivity, and scalability never seen before in the field of cell concentration measurement [10,11,12]. Furthermore, as the need for highly sensitive, rapid, and cost-effective cell concentration measurement methods is constantly growing, microfluidic devices are playing a pivotal role in advancing research, diagnostics, and therapeutics [13,14,15]. Considering their suitability for automation, which allows for high-throughput analysis of cell concentrations, they are particularly useful in clinical and research settings where large datasets need to be generated rapidly [16,17].

Microfluidic devices for measuring cell concentration use various principles and techniques to accurately quantify the cell concentration in a given sample [18], such as *flow cytometry* [19,20], *impedance spectroscopy* [21,22,23], *digital microfluidics* [24,25,26,27,28,29], *acoustic-based microfluidic devices* [30,31], and *microscopy and image analysis* [32,33]. These techniques are designed to be highly sensitive and efficient, making them helpful tools in fields such as biology, medicine, and biotechnology. The working principle, advantages, and drawbacks related to the main principles and techniques employed in these devices are reported in Table 1. Even though all the abovementioned techniques have already been exploited in the literature for the stated purpose, some cons can be derived from their implementation. For example, the *flow cytometry* techniques are quite invasive because they are label-based and require the use of various dyes, while the *impedance-spectroscopy*-based approach needs electrical components integrated in the device in addition to the use of a conductive medium, which often differs from the cells’ culture fluids. Developing and operating *digital microfluidic* systems can be complex and costly due to the need for specialized equipment, including microcontrollers, electrodes, and high-resolution cameras. Next, *acoustic-based microfluidic devices* can be complex to design and costly to fabricate since creating the necessary transducers and acoustic waveguides can require specialized expertise and equipment. Moreover, they are sensitive to environmental factors such as temperature, humidity, and air quality; so, variations in these factors can affect the accuracy and reproducibility of the measurements.

To overcome all of the disadvantages discussed up to now, the use of the *optical detection* methods for cell concentration monitoring offer several advantages [34,35,36,37] such as: (i) high sensitivity, which is essential for accurately measuring low concentrations of cells; (ii) real-time or near-real-time monitoring of cell concentrations, particularly valuable for dynamic processes; (iii) label-free detection, by simplifying sample preparation and reducing potential artifacts from labeling; (iv) a noninvasive approach, which is essential for preserving cell integrity during measurements; (v) the use of a low sample volume, which is useful when working with limited or precious samples, even by reducing reagent consumption; and (vi) integration with microfluidics, allowing for the efficient, miniaturized, and high-throughput analysis of cell concentration in a lab-on-a-chip format. Thus, micro-optofluidics is an extension of microfluidics, which enhances the capabilities of fluid manipulation with the integration of optical components, such as lenses, waveguides, and detectors [38,39,40]. While both microfluidics and micro-optofluidics share the focus on small-scale fluid manipulation, interdisciplinarity, and lab-on-a-chip applications, micro-optofluidics stands out due to its integration of optical components, allowing for advanced analytical capabilities and diagnostic purposes involving light-based interaction with fluids. Due to its suitability for a wide range of measurements and applications, the *optical one* is generally applicable for two-phase flow control and monitoring. The term two-phase flow covers two immiscible fluids, one dispersed in the other that circulates within the same microsystem, i.e., in the context of microfluidic devices within the same microchannel. According to its definition, the two-phase flow may be formed by: immiscible liquid–liquid, gas–liquid, and microparticles suspended in a liquid [41,42]. By following the latter discussed detection approach, a microfluidic device can incorporate a detection system, such as a photodetector or a spectrophotometer, to measure the light absorbance or scattering of cells in the sample and relate the degree of absorption or scattering to the concentration of cells suspended in a fluid.

In this work, a biocompatible micro-optofluidic (MoF) device for two-phase flow control and monitoring, relying on an optical detection approach, was manufactured by using the Projection Microstereolithography (PμSL) 3D printing technique. By means of the developed MoF device with the selected manufacturing technology, two different two-flow phase processes were successfully monitored: an (i) air–water bi-phase flow and (ii) a two-phase mixture composed of micrometric cells suspended on a liquid solution. In this way, a step forward with respect to more traditional techniques for cell concentration evaluation was taken. Hence, commonly, fabricating microfluidic devices for biomedical applications can be challenging because the use of biocompatible materials, also showing chemical compatibility and optical transparency, is required. Additionally, very high precision requirements must be satisfied since the tolerances for microfluidic device fabrications are extremely tight: even small variations in channel dimensions, surface properties, or alignment can lead to significant operational issues. Thus, achieving high precision in fabrication processes is essential. In the past, techniques such as deposition, microfabrication photolithography, and etching processes were used to manufacture microfluidic devices [43,44,45,46]. However, these fabrication techniques involve time-consuming procedures that also require cleanrooms and have high costs of production because expensive raw materials are used, such as quartz, silicon, and glass. Moreover, the aforementioned strategies do not allow the feasibility of a complex channel’s geometry to be achieved and the required high level of precision to be satisfied. These drawbacks were overcome with the advent of 3D printing technologies, which allow for low-cost and simple fabrication processes (few steps required) that are also compatible with mass production [47,48,49,50,51,52,53,54]. Among the different existing *3D printing (3DP)* techniques, the *fused filament fabrication (FFF)*, *inkjet-based 3DP* (i.e., *PolyJet* and *MultiJet*), and *vat polymerization (VP)* methods (i.e., stereolithography and digital light processing) were exploited to manufacture polymeric microfluidic devices, such as bioreactors for real-time biological analysis or analytical systems [50,51,52,53,54], meeting a precise design and geometry. In line with the review of G. Gonzalez et al., by using the *FFF 3DP* technology, the smallest microfluidic channel achieved is equal to 40μm [55], while it is equal to 54μm for *PolyJet/MultiJet 3DP* techniques [56] and 18μm by using *VP* methods [54]. All of these *3DP* techniques have pros and cons for microfluidic device manufacturing. Focusing on *FFF*, it is affected by a limited precision, with its resolution limited by the nozzle’s diameter [57,58]. Moreover, the materials typically used with this technique are characterized by a limited optical transparency, so result in being useless for optical or image detection. Finally, the surface finish achieved through this technique, i.e., the surface roughness (ranging between 9 and 40μm), is not low enough to ensure a hydrodynamic stability of the fluid within the microchannels. The latter drawback is also typical for the *inkjet-based 3DP* methods, even though pros such as fast manufacturing times and the opportunity to realize multimaterial devices are guaranteed [59]. Conversely, a high printing resolution together with a good surface finishing of about 0.4 and 2μm is achieved by exploiting the *VP 3DP* technology. In addition, a strong advantage related to the latter method for microfluidic device fabrication consists of the chance to directly realize microfluidic channels, within the device in one piece, without using supporting material [60,61]. The latter is usually needed to successfully carry out the manufacturing procedure of a part with complex geometries (holes or overhang), thus avoiding collapsing or wearing phenomena, although at the end of the 3DP procedure, it must be removed. However, in the field of microfluidics, the support material removal in micrometric channels within the 3D printed structure can be very labor intensive, time-consuming, or even impossible since it is located in difficult-to-reach regions within the part, i.e., enclosed microchannels [62,63]. Furthermore, residual sacrificial material attached on the microchannel’s walls can cause surface properties’ alteration (i.e., roughness and wetting ability), which may lead to hydrodynamic instability within the device. In other cases, when the support material removal implies the use of a chemical solution, this process can lead to damage to the structure itself since it has a very small size (micrometric). To overcome this issue, the “*additive assembly methodology*” could be exploited, which means designing the device in separate parts (two unenclosed parts) that can be assembled as soon as the manufacturing process is completed. This approach has already been used [64]. In this way, it is possible to avoid the use of support material within the microfluidic channel by appropriately orienting the part on the printing platform. However, as soon as the assembly is realized, fluid leakage problems can occur since a total bonding that is durable over time is difficult to achieve. A similar problem is also related to the *master–slave microfabrication 3DP* approach, commonly used to realized polydimethylsiloxane (PDMS) microfluidic devices [65], where the 3D printing is used to realize 3D printed mold, and further used to pour the PDMS and fabricate the final device [66]. Even though it is an easy, low-cost, and one-step process without directly handling hazardous chemicals, the final assembly procedure of different parts, with the aim to realize the final device, involves issues such as no permanent bonding being achievable and fluid leakage.

In this work the PμSL 3D printing approach was selected because it allows for the creation of monolithic microfluidic structures, meaning that the entire device, including channels, chambers, and other components, can be fabricated in one piece. Thus, this eliminates the need for labor-intensive assembly processes and reduces the risk of leakage or contamination at interfaces. In addition, with the used 3D printer being a 10μm series machine, this allowed a level of precision and accuracy to be achieved for the device that delivers the most challenging micro parts at production quality [67,68]. Next, the chosen manufacturing technique offered the ability to use a biocompatible material, named *BIO resin*, allowing for the development of an MoF device for the cells’ application. To assess the suitability of the selected biocompatible transparent resin for an optical detection relying on the selected working principle (absorption phenomenon), a comparison between a two-phase flow process detected inside a previously fully characterized micro-optofluidic device made of a nonbiocompatible high-performance resin (HTL resin) [66] and the same made of the biocompatible one (BIO resin) was carried out first in this work. In this way, it was possible to highlight the main differences between the two different resin grades, which were further justified with proper chemical analysis of the used resins and their hydrophilic/hydrophobic nature via static water contact angle measurements. Furthermore, to check the MoF device’s capability for cell concentration monitoring, a wide experimental campaign was performed in different operative conditions, i.e., testing the detection of different concentrations of eukaryotic yeast cells of *Saccharomyces cerevisiae* (with a diameter of 5 μm) suspended on a PBS (phosphate-buffered saline) solution. PBS is a nontoxic solution used to suspend cells. Its use, unlike water, helps to maintain cells’ physiological conditions by preventing their swelling or shrinkage due to osmosis [69]. Furthermore, its adoption with optical detection methods minimizes light scattering by also reducing optical distortions and background noise, leading to a clearer and more accurate optical measurement. This is due to there being similar refractive index values between biological samples, including yeast cells ($r_{Yeast}=1.5$) [70] and PBS ($r_{PBS}=1.3$) [71], rather than water ($r_{Water}=1.0$) that can result in increased scattering and aberrations phenomena in optical detection. Finally, since the here-proposed device integrates both microfluidic and micro-optical components, it is suitable to run real-time analysis of a low sample volume by using a noninvasive and label-free optical detection approach. In this sense, the MoF device presented in this study represents a powerful and reliable diagnostic tool realized by overcoming common manufacturing technical and real-time analysis challenges of the microfluidic field.

This paper is organized as follows. Section 2 presents the materials used and the methods followed methods to design and manufacture the MoF device together with its working principles (Section 2.1 and Section 2.2). The used characterization techniques are presented in Section 2: in detail these are: the chemical characterization of the used material (Section 2.3 and Section 2.4); the quality monitoring of the achieved accuracy for the MoF device (Section 2.5); and the experimental setup implemented and used to fully characterize the MoF device (Section 2.6). The post-processing procedure of the acquired optical signals is explained in Section 2.7, while the experimental campaigns that were carried out are presented in Section 2.8, Section 2.9 and Section 2.10. The results regarding the device characterizations are discussed in Section 3. Finally, the conclusions and future research directions are reported in Section 4.

## 2. Materials and Methods

### 2.1. Materials

The MoF device was made of a photocurable biocompatible resin, named BIO resin and commercialized by *Boston Micro Fabrication Materials Technology Co., Ltd.* (BMF, Maynard, MA, USA). It is a yellow-grade resin suitable for sterilization processes, having a Heat Distortion Temperature (HDT) of $85.7{\;}^{\circ }$C at a pressure of 0.45 MPa. Its estimated refractive index value is equal to $1.6859\pm 1.73\times 10^{-4}$ [72]. According to its TDS (technical data sheet) https://bmf3d.com/wp-content/uploads/2023/02/BMFBIOResinDatasheet_022823.pdf (accessed on 13 October 2023), it finds application in the health and biomedical engineering field since it has passed diverse ISO 10993 https://bmf3d.com/wp-content/uploads/2023/02/BMFBIOResinDatasheet_022823.pdf (accessed on 8 November 2023), biocompatibility tests, i.e., in vitro hemolysis, cytotoxicity, pyrogenicity, toxicity, skin irritation, and sterilization. The BIO resin was used to manufacture the MoF device via Projection Microstereolithography (PμSL) 3D printing on a microArch^®^S140 3D printer (BMF, Maynard, MA, USA), which is characterized by an ultra-high *XY*-optical resolution equal to 10μm, a *Z*-resolution (layer thickness) ranging between 10 and 40μm, and a building volume of 94×52×45mm^3^.

Eukaryotic yeast cells of *Saccharomyces cerevisiae* (with a diameter of 5 μm) suspended on a PBS (phosphate buffered saline) solution were used as biological samples to assess the capability of the developed MoF device to perform cell concentration monitoring analyses.

### 2.2. MoF Device: Design, Manufacturing, and Working Principle

The micro-optofluidic device developed in this experimental work was, at first, designed using Autodesk^®^Fusion 360 (Autodesk Inc., San Rafael, CA, USA). Next, it was directly manufactured as a single piece through a highly innovative microprecision 3D printing technique, named Projection Microstereolithography (PμSL). It is a variant of the standard SLA (stereolithography) 3D printing technique, which was modified in accordance with the DLP (digital light processing) working principle. In fact, while the standard SLA technique uses a UV laser to cure the liquid resin, which is oriented using mirrors driven by a galvanometer system through the building volume, the PμSL technology was developed as an alternative to the DPL 3D printing technique, using a projector as a light source rather than a laser and by also considering two additional key elements. Firstly, a high resolution is achieved by placing a high-precision lens in between the bath of resin and the projector. Secondly, the light source moves in the XY plane, allowing large or multiple parts to be printed by projecting the same mask multiple times while maintaining a high resolution. In this way, it is possible to achieve an XY resolution down to 2μm, a minimum feature size of 10μm, and a dimensional tolerance as high as ±10μm. Thus, PμSL enhanced both the standard SLA and DLP performance. Indeed, the SLA method has an XY resolution of 50 µm, a minimum features size of 150μm, and an overall tolerance of ±100μm, while the DLP one achieves an XY resolution of 25–50 μm, a minimum feature size of 50–100 μm, and an overall tolerance of ±75μm. Finally, the PμSL technique exploits a top-down approach, which permits the use of support material to be either minimized or avoided.

The selected printing settings to manufacture the MoF device, in addition to the following washing and post-processing procedure, are reported in Table 2.

**Figure 1:**
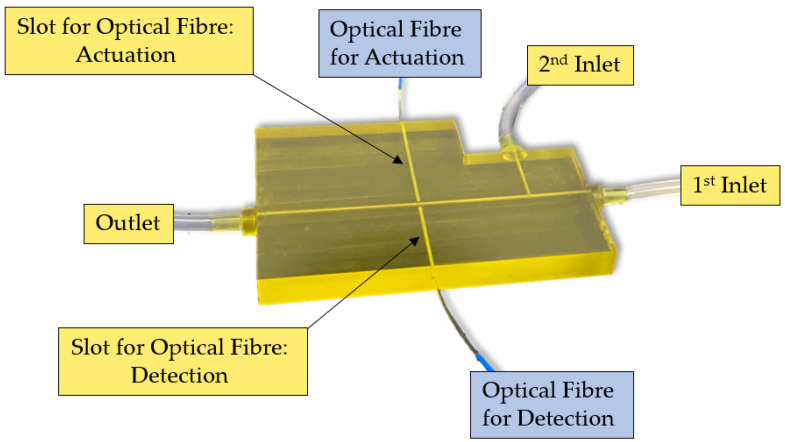
Three-dimensional printed MoF device. It presents: (i) two connected microchannels to form a T-junction for the two-phase flow formation; (ii) two micrometric slots for optical fibers insertion; and (iii) two inlets to introduce the two fluids within the microfluidic channel and one outlet to convey the fluid out.

**Figure 2:**
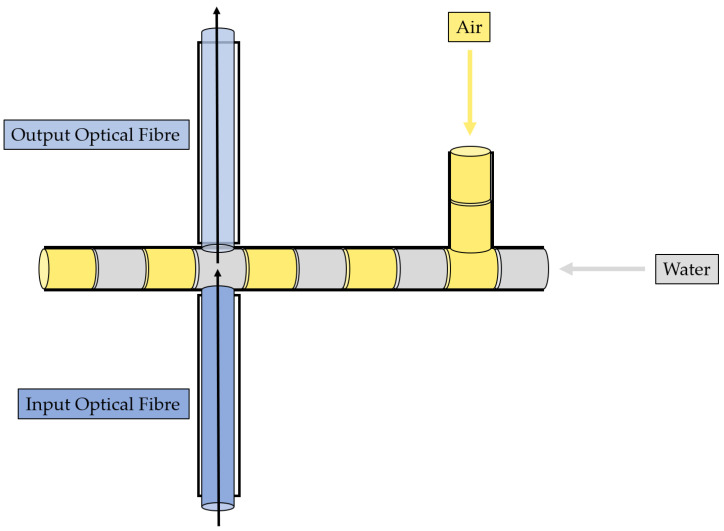
Working principle of the MoF device for immiscible gas–liquid two-phase flow detection.

**Figure 3:**
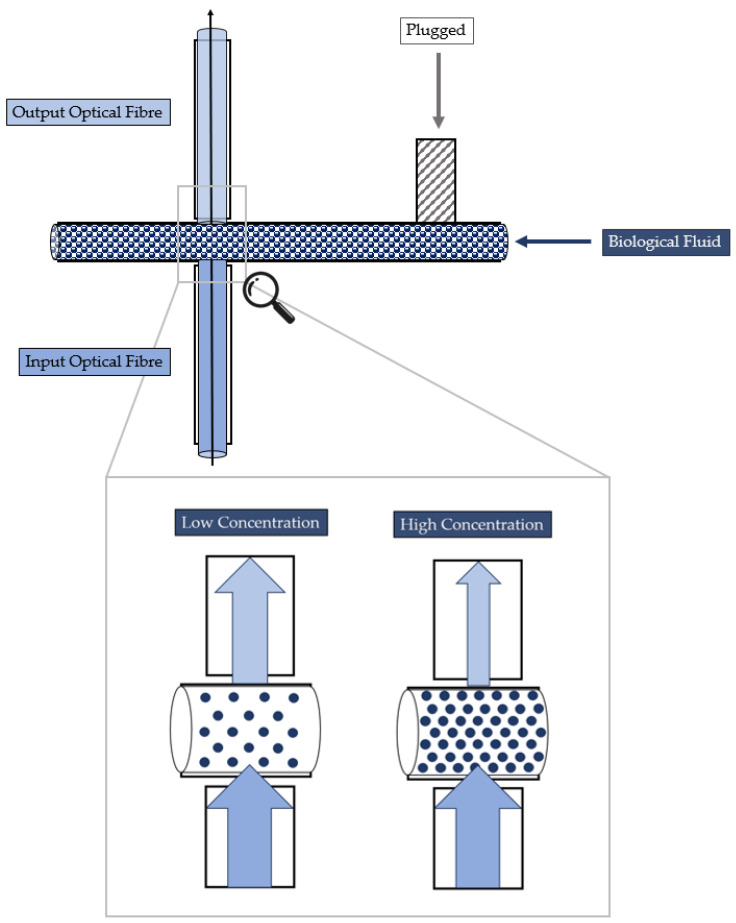
Working principle of the MoF device for cell concentration monitoring.

Its geometry was developed by carrying proper simulations, and the details are provided in a previous study [37]. The device is characterized by two connected microchannels to form a T-junction for the two-phase flow formation. Furthermore, far enough from the T-junction that forms the two-phase flow to guarantee its hydrodynamic stabilization, there are two micrometric slots designed for optical fibers’ insertion, which are laid out orthogonally with respect to the main microfluidic channel. There are two inlets to introduce the two fluids within the microfluidic channel and one outlet to convey the fluid out. All of them were realized according to the parallel (in-line) approach thanks to the selected manufacturing technique. According to the latter approach, the inlet and the microchannel are arranged in a way so that they are parallel to each other and are aligned along the same axis. The 3D printed device is shown in Figure 1. The MoF device can be used for:

(i) *Immiscible gas–liquid two-phase flow detection*—its working principle is reported in Figure 2 and relies on the absorption phenomenon. In fact, depending on the fluid’s refractive index value, its interaction with the incident laser beam determines a different nature of light transmission. Thus, in turn, the acquired optical signal has a different amplitude depending on the fluid with which it is interacting at a precise moment. More deeply, the acquired optical signal has a square wave shape, characterized by two levels corresponding to each fluid making up the two-phase flow;
(ii) *Cell concentration monitoring*—its working principle is reported in Figure 3 and exploits the cell–light interaction linked to the different cell concentration. The higher the concentration, the greater the number of cells that interact with the light, which, in turn, causes the light’s back-scattering. Thus, as consequence, the beam does not reach the outgoing optical fibre. Consequently, with increasing concentrations, there is a corresponding decrease in the measured levels of light intensity.

### 2.3. Surface Characterization: Static Water Contact Angle and Roughness Measurements

To evaluate the behaviour at the solid–liquid interface, i.e., to determine if the selected photocurable resin for the MoF manufacturing is either a hydrophilic or hydrophobic surface, static water contact angle (θ) measurements were run. This kind of analysis is crucial because the nature of the wettability of the microchannel wall’s solid surface may influence the exchange momentum of the fluid with the solid surface itself at the atomic scale, so causing related variation in the hydrodynamic processes [73,74]. The experiments were carried out using a Lite Optical Tensiometer TL100 with an accuracy of $\pm 3^{\circ }$. The tests were performed by depositing on the resin’s surface, firstly, 5μL of Milli-Q water drop (with a resistivity of 18.2 MΩ at $25{\;}^{\circ }$C), at room temperature and in air atmosphere. Secondly, the parameter θ was measured in correspondence with the two-dimensional projection of the droplet. The measurements were replicated on N=5 different samples to achieve statistically reliable results.

According to [75], the surface roughness is subject to changes when moving from macroscale to microscale. In fact, the validity of classical theory is not applicable in both cases. Therefore, it is crucial to investigate such a property in our scenario. The surface roughness measurements were carried out using Atomic Force Microscopy (AFM). An AFM NTEGRA, NT-MDT (Zelenograd, Russia) was used in semi-contact mode, with a rate of 0.5 Hz. Moreover, a tip ETALON series (NT-MDT, Zelenograd, Russia) characterized by a resonant frequency of 140±10% kHz was used. The software used to evaluate the surface roughness was Image Analysis of Nova Px (v.3.4.0) and a 5×5μm^2^ area was investigated. N=5 replications were collected to achieve statistically reliable results.

### 2.4. Cross-Linking State of BIO Resin: FT-IR ATR Analysis and Refractive Index Estimation

In 3D printed objects fabricated via light-based 3D printing techniques, the issue of nonhomogeneous photocuring conversion, due to a nonlinear conversion of photo-polymerization and nonmonotonic spatial monomer-to-polymer conversion of material phenomena, tends to cause nonuniform network properties, such as density, permeability, and refractive index [76]. However, the MoF device here developed should be characterized by a uniform refractive index value throughout its structure to meet the working principle it was designed for.

**Figure 4:**
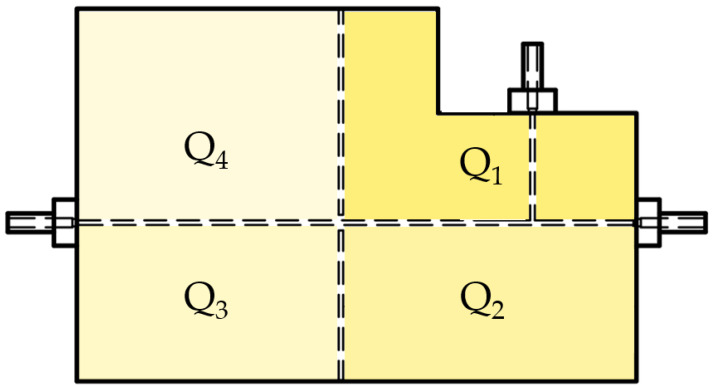
MoF device’s investigated quadrants $Q_{i}$, with i=4, with the FT-IR ATR analysis and the refractive index value estimation.

To check if the selected manufacturing technique, i.e., the (PμSL), allowed a device to be obtained that was made of a resin characterized by a uniform cross-linked network, Fourier-transform infrared attenuated total reflectance (FT-IR ATR) spectroscopy was used as the investigation technique to monitor the cross-linking state of the photocurable resin at different investigation points of the 3D printed device’s structure. This approach was previously used in the state-of-the-art [76,77]. As a part of the investigation, four different quadrants of investigation, $Q_{i}$ with i=1,…,4, were analyzed, as shown in Figure 4. Subsequently, a refractive index estimation ($\hat{R}$) of each investigated quadrant $Q_{i}$ was conducted to assess the achieved network’s homogeneity. This assessment is crucial to ensure that no variations are present in the network. The refractive index values were estimated by following a novel method already proposed by the authors [72]. The FT-IR analyses were run using a Perkin Elmer Spectrum 100 UATR (Waltgam, MA, USA) in attenuated total reflectance (ATR) mode and were acquired with 32 scans with a resolution of 4 cm${}^{-1}$ within the range (4000;650)cm${}^{-1}$.

### 2.5. Three-Dimensional Printed Microchannel: Quality Monitoring

**Figure 5:**
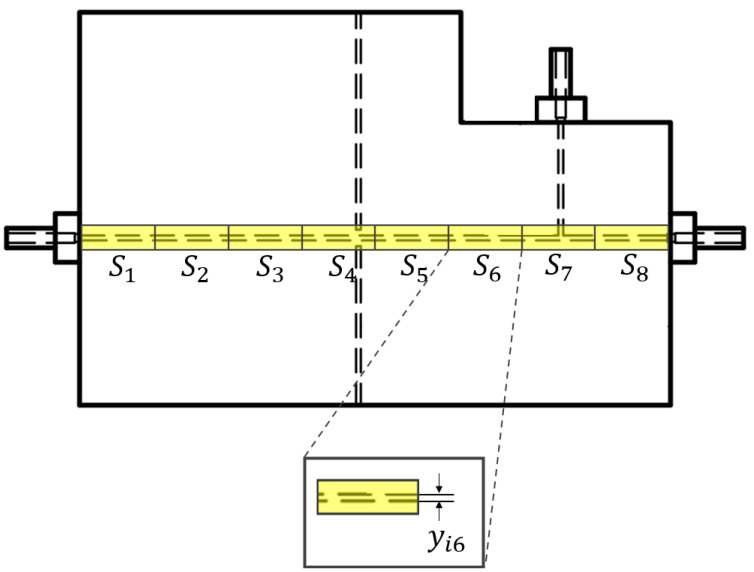
Investigated sections $S_{j}$, with j=1,…,s and s=8, of the MoF device for assessing the quality of the channel’s width $y_{ij}$.

The hydrodynamic process of the designed, manufactured, and tested MoF device strongly depends on its channel width stability. Thus, to prove the quality of manufacturing, which in turn derives from the selected 3D printing technique and the photocurable resin, a profile monitoring was run to check that the channel’s width measurements are consistent with the declared manufacturing process accuracy (10μm). The observations of the channel’s width $y_{ij}$ were collected by splitting the microfluidic channel into s=8 different sections ($S_{j}$) with j=1,…,s, as illustrated in Figure 5. For each investigated section, i=5 observations were collected. To run the channel’s width measurements, appropriate images were acquired for each investigated section $S_{j}$ by using a microscope (B-380, OPTIKA, Ponteranica, BG, Italy ), including the objective lens and the hardware components coupled with a CCD camera (CS165MU, Thorlabs, Newton, NJ, USA) with a resolution of 1440×1080 px (pixel size of 3.45μm, square). In detail, each channel’s width $y_{ij}$ parameter was measured using the software ImageJ (v.1.53t) https://imagej.net/ij/ (accessed on 10 November 2023). Next, the average, the standard deviation, and the standard error were evaluated for the investigated parameter and compared with the declared accuracy specification defined for the selected manufacturing process.

### 2.6. Experimental Setup: Two-Phase Flow Process and Cell Concentration Monitoring

**Figure 6:**
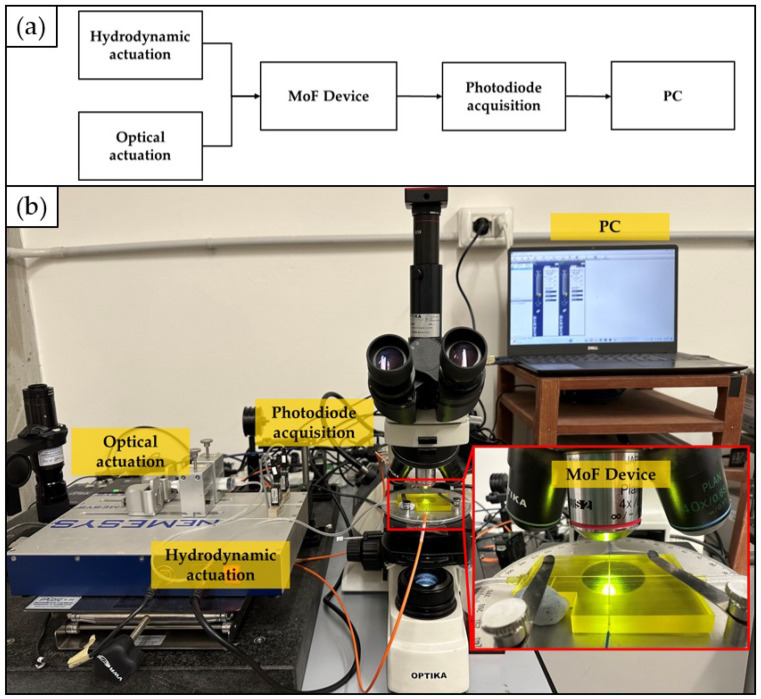
Experimental setup: (**a**) block scheme; (**b**) real picture.

The experimental setup used to obtain the two-phase flow and to monitor the cell concentration through the micro-optofluidic device is schematically depicted in Figure 6a. In particular, it is composed by (i) the hydrodynamic actuation system to inject the fluid samples inside the device; (ii) the optical actuation system represented by the light source introduced inside the device through an input optical fiber; (iii) the micro-optofluidic device; (iv) an optical detection system through a photodiode connected to the output optical fiber, and (v) a PC for the measurement acquisition through dedicated software. Additionally, the real experimental setup is shown in Figure 6b.

Two classes of experiments were conducted. Firstly, deionized water and air were pumped concurrently through the two inlets of the T-junction geometry to create the two-phase flow through the micro-optofluidic device. Specifically, two different syringe pumps (neMESYS), the former filled by air and the latter filled by deionized water, were connected to the two inlets to inject the samples of fluid in the micro-optofluidic device. Secondly, a single syringe pump was connected to an inlet of the device and was used to inject the solution of yeast cells suspended in PBS in the main channel of the micro-optofluidic device. In this case, the second inlet was properly plugged. A laser system (NovaPro 660–125, RGB Lasersystems, Kelheim, Germany) with an emission wavelength equal to 600 nm was connected through an SMA connector to a 365 μm diameter input optical fibre, coupled to the device for the optical actuation. Moreover, the micro-optofluidic device was coupled with another 365 μm diameter output optical fiber, required for the optical detection. The latter was connected to a photodiode with a gain of 40 dB (PDA100A, 144 Thorlabs, Newton, NJ, USA), able to measure the light intensity variation. A PC oscilloscope (Picoscope 2204A, Pico Technology, Cambridgeshire, UK), with a sampling frequency of 1.5 kHz, was used to acquire the detected optical signals.

In addition, a microscope (B-380, OPTIKA, Italy), including the objective lens and the hardware components, was used to retain and align the optical elements and the device. A CCD camera (CS165MU, Thorlabs) with a resolution of 1440 × 1080 px (pixel size of 3.45 μm, square) was coupled with the microscope. The CCD camera was connected through a USB connection with a PC for the frames’ acquisition and the subsequent analysis phase in the dedicated software platform. A magnification of 4× (PLN, Olympus) was set to scale up the channel images and increase the image resolution. The microscope’s inclusion in the setup was crucial to obtain high-resolution frames of the channels, essential for the 3D printed channels’ quality monitoring described in detail in Section 2.5.

### 2.7. Acquired Optical Signals’ Post-processing and Investigated Responses’ Calculation

In line with the working principle explained in Section 2.2 for the immiscible gas–liquid two-phase flow, the acquired square wave signal is characterized by a lower level, associated to the air slug ($n_{Air}=1.0$), and a higher level corresponding to the water slug ($n_{Water}=1.3$), while the transient phase between the fluids is marked by a peak.

The acquired signals were post-processed in MATLAB (v.R2023b, MathWorks^®^). In detail, a low-pass filter with a cut-off frequency of 40 Hz was applied to remove high frequency harmonics. Next, a smoothing procedure was used to eliminate the noise from the signal and reveal the main square wave pattern. From the post-processed signals, it was possible to find the time interval related to the higher water level ($T_{w}$) and the lower air level ($T_{a}$).

Additionally, the detected optical signal was investigated in the frequency domain. The fundamental harmonic was evaluated extracting the highest peak from the spectrum (*f*), and from the reciprocal of this parameter, the corresponding period (*T*) was calculated.

Two different responses were considered from the detected optical signal square-wavelike: the voltage peak to peak difference ΔV and the mean period *T* related to a complete two-phase flow passage.

The first one was evaluated as follows. The water (higher) and the air (lower) levels were collected in time frames obtaining a single sample of $V_{w}\left(i\right)$ and $V_{a}\left(j\right)$ observations, for $i=1,\ldots ,N_{w}$ and $j=1,\ldots ,N_{a}$. Here $N_{w}\in \left(10^{3},10^{4}\right)$ and $N_{a}\in \left(10^{3},10^{4}\right)$. Then, for each sample $V_{w}\left(i\right)$ and $V_{a}\left(j\right)$, both the mean values (${\bar{X}}_{w}$ and ${\bar{X}}_{a}$) and the standard deviations ($s_{w}$ and $s_{a}$) were calculated. Since the two samples $V_{w}\left(i\right)$ and $V_{a}\left(j\right)$ have a huge number of observations, we can assume that their sample means are approximately normally distributed with estimated parameters ${\bar{V}}_{w}∼N\left({\bar{X}}_{w},\frac{{s_{w}}^{2}}{N_{w}}\right)$ and ${\bar{V}}_{a}∼N\left({\bar{X}}_{a},\frac{{s_{a}}^{2}}{N_{a}}\right)$. Under this assumption, the voltage difference is defined as:

$$\Delta V={\bar{V}}_{w}-{\bar{V}}_{a}$$

with ΔV a normal random variable with distribution $\Delta V∼N\left({\bar{X}}_{w}-{\bar{X}}_{a},\frac{{s_{w}}^{2}}{N_{w}}+\frac{{s_{a}}^{2}}{N_{a}}\right)$.

The second response was calculated as follows:

$$T=\frac{1}{f}=<T_{w}>+<T_{a}>$$

Moving on the working principle explained in Section 2.2 for the cell concentration monitoring, the acquired optical signal has a monotonic trend. The post-processing routine followed in this case was similar to the one described above, i.e., by performing the filtering and smoothing steps in MATLAB (MathWorks^®^), followed by the determination of the mean value ($S_{ph}$) and standard deviation.

### 2.8. Two-Phase Flow Process: Experimental Campaign BIO Device

To investigate the immiscible gas–liquid two-phase flow process for the BIO device, a replicated general factorial design was run. The factors (independent variables) investigated in the experimental design were:

- *Laser input power (factor A)*—Quantitative factor varied at three levels (a = 3) corresponding to {1, 3, 5} mW;
- *Fluid flow rate (factor B)*—Quantitative factor varied at three levels (b = 3) corresponding to {0.1, 0.2, 0.3} mL/min.

The number of replications was set to n = 3, for a total of N = a × b × n = 27 experimental runs. The experimental runs were not completely randomized, but the experiments were divided into 3 different blocks, each of them corresponding to a different replication (n=1,2,3). The division by blocking should balance out the effect of the replications with the aim to eliminate its influence on the analysis. The experimental plan is reported in Table 3. The following responses (dependent variables) were considered for investigation by the experimental plan: the *voltage difference* (ΔV), which was considered to show the device’s ability to discriminate between two different fluids making up the two-phase flow (air–water), and the *mean period* (*T*) associated with a complete two-phase flow passage. The statistical significance of each factor and their possible interaction were examined using an Analysis of Variance (ANOVA) table once the values of each response were determined.

### 2.9. Two-Phase Flow Process: Experimental Campaign and Comparative Analysis between BIO Device and HTL Device

Once the full characterization for the immiscible gas–liquid two-phase flow process was carried out for the BIO device, a comparative analysis was performed versus an already developed and investigated MoF device (HTL resin) [66], which presented quite good performance and very low repeatability error. However, the HTL resin of the MoF device is nonbiocompatible; thus, not properly suitable for cell applications. This comparison allowed an assessment of whether the novel BIO device has similar performance to the MoF device and is affected by low repeatability error. For this purpose, a replicated general factorial design was carried out. Three design factors (independent variables) were considered for the two-phase flow process comparative analysis between BIO and HTL devices:

- *Laser input power (factor A)*—Quantitative factor varied at two levels (a = 2) corresponding to {1, 5} mW;
- *Fluid flow rate (factor B)*—Quantitative factor varied at three levels (b = 3) corresponding to {0.1,0.2,0.3} mL/min;
- *Material (factor C)*—Categorical factor varied at two levels (c = 2) corresponding to {HTL, BIO}.

The number of replications was set at n = 3, for a total of N = a × b × c × n = 36 experimental runs. The experimental runs were not completely randomized, but the experiments were divided into 3 different blocks, each of them corresponding to a different replication (n=1,2,3). The division by blocking should balance out the effect of the replications with the aim to eliminate its influence on the analysis. The experimental plan is reported in Table 4. The investigated responses are the *voltage difference* (ΔV) and the *mean period* (*T*) associated to a complete two-phase flow passage. After the responses were measured, an Analysis of Variance (ANOVA) table was used to identify the statistical significance of each factor and any potential interaction.

### 2.10. Cell Concentration Monitoring: Experimental Campaign (BIO Resin)

After having proved the reliability for the BIO device throughout the considered operative range for the immiscible gas–liquid two-phase flow process, two different flow rate values were investigated for cell concentration detection, i.e., {0.05, 0.1} mL/min, to find a well-dispersed condition for the cells within the PBS solution. At this stage, it is crucial to find a trade-off for the hydrodynamic process avoiding either the cells’ precipitation on the channel’s floor (when FR is too low) or to have a too fast flow of particles (i.e., cells) causing a noisy acquired optical signal, which is due to many scattering phenomena (when FR is too high) [32]. To optimize the working condition of the BIO device for cell concentration monitoring, a properly general factorial design was studied, where three design factors (independent variables) were considered:

- *Laser input power (factor A)*—Quantitative factor varied at three levels (a = 3) corresponding to {1, 3, 5} mW;
- *Concentration of yeast cells (factor B)*—Quantitative factor varied at two levels (b = 4) corresponding to {0, 10^6^, 10^7^, and 10^8^} in 10 mL PBS;
- *Fluid flow rate (factor C)*—Quantitative factor varied at two levels (c = 2) corresponding to {0.05, 0.1}mL/min.

The number of replications was set at n = 1, for a total of N = a × b × c × n = 24 experimental runs, which were completely randomized. The experimental plan is reported in Table 5. The investigated response was the average voltage value ($S_{ph}$). Once an optimal range for the power and flow rate was identified, to investigate the reliability of the device, a new replicated general factorial design was investigated. It is described in detail in Section 3.3.

## 3. Results and Discussion

### 3.1. Immiscible Gas–Liquid Two-Phase Flow Process

**Figure 7:**
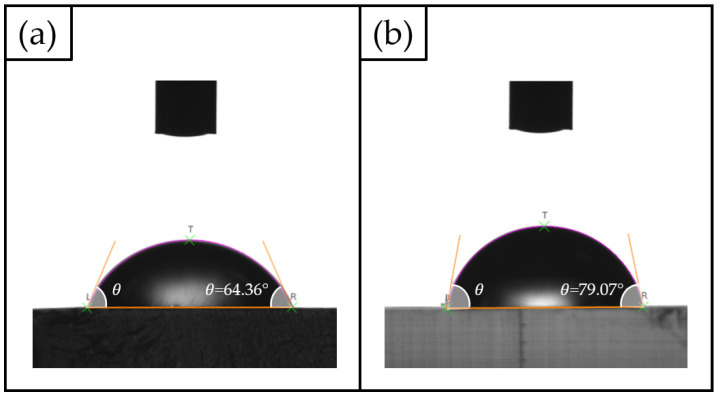
Static water contact angle results: (**a**) HTL and (**b**) BIO resins.

Water contact angle measurements (see Figure 7) revealed that a slight difference exists for the new BIO resin when compared to the already tested HTL one. While the former presented a θ value equal to $79.07\pm 0.85^{\circ }$, for the latter the angle was equal to $64.36\pm 1.63^{\circ }$. Even though the two investigated materials present a hydrophilic behaviour $\left(\theta <90^{\circ }\right)$, the HTL one is stronger.

**Figure 8:**
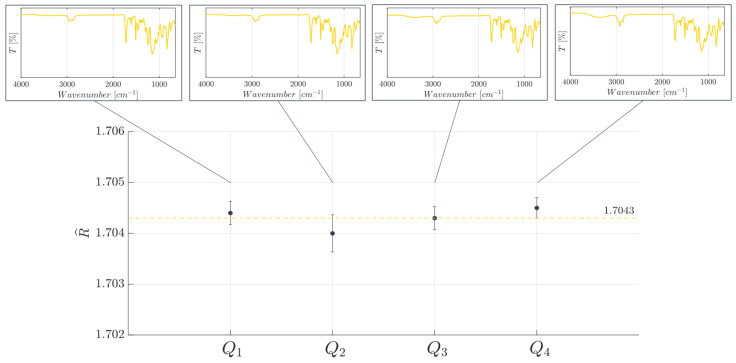
Estimated refractive index value ($\hat{R}$) dependence on the BIO resin’s chemical uniformity investigated on four different quadrants $Q_{i}$ of the MoF device. The yellow dotted line represents the overall estimated mean value.

Next, after focusing on the cross-check between the FT-IR ATR and the refractive index value estimation carried out on the four investigated quadrants for the selected BIO resin, the obtained results are reported in Figure 8. For each investigated quadrant $Q_{i}$, a homogeneous phase can be observed, which is confirmed with all the spectra characterized by the same peaks (see Figure 8). This result demonstrates a homogeneous photocuring process occurred during the 3D printing process of the manufactured device. Further results demonstrating the full curing of the used system (BIO resin) can be associated to the absence of a peak at 1644 cm${}^{-1}$, generally linked to the double bonds of acrylates. The highly intense band from 1060 to 1190 cm${}^{-1}$ is associated to the stretching of the C−O−C bond typical of the ester group in polyacrylates. Moreover, as a result of the cross-check analysis, the assessed homogeneous photocuring condition allowed a steady estimated refractive index value ($\hat{R}$) to be achieved throughout the device’s surface, i.e., for each $Q_{i}$, as shown from the scatter plot in Figure 8. An overall mean value for the estimated parameter ($\hat{R}$) is $1.7043\pm 1.2779\times 10^{-4}$, and this result is consistent with our previous work [72].

**Figure 9:**
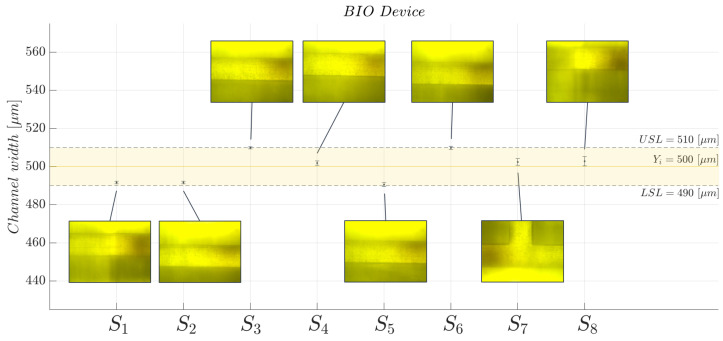
Scatter plot for the BIO device’s channel width determination as function of each investigated section $S_{j}$, with j=1,…,8.

Finally, regarding the quality control analysis conducted for the BIO device channel’s width, no significant geometric anomalies able to cause the flow instability phenomenon within the microchannel were found; see each investigated frame in Figure 9. Indeed, focusing on the scatter plot reported in Figure 9, where the measured channel’s widths as functions of each investigated section $\left(S_{j}\right)$ are reported, all the measured mean values fluctuate around the nominal design channel’s width $(Y_{i}=500\;\mu$m) (yellow dotted line) and fall within the *Lower Specification Limit (LSL)* and the *Upper Specification Limit (USL)*. This result confirmed a measured accuracy specification, which expresses how the measured value $\left(y_{ij}\right)$ differs from the nominal design channel’s width $(Y_{i}=500\;\mu$m), of 7.01±0.56μm that is consistent with the value declared by the BMF company.

### 3.2. BIO Device: Characterization Results

#### 3.2.1. BIO Device

**Figure 10:**
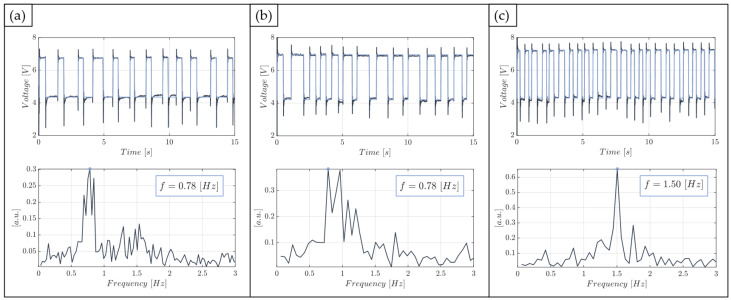
Acquired optical signals in the time domain (upper panel) and in the frequency domain (lower panel) with P=3 mW at different flow rate conditions: (**a**) FR=0.1 mL/min, (**b**) FR=0.2 mL/min, and (**c**) FR=0.3 mL/min.

Figure 10 shows the optical signals in the time domain (upper panel) and the corresponding spectra (lower panel) associated to the operative condition with P=3 mW and different flow rates, i.e., (a) FR=0.1 mL/min, (b) FR=0.2 mL/min, and (c) FR=0.3 mL/min. For the sake of brevity, only the mentioned working conditions are shown; detailed results concerning the other investigated scenarios are available upon request from the authors.

From the square wave optical signals, it is possible to differentiate each fluid of the two-phase flow. More precisely, the three time domain signals clearly show the distinction between the higher water level and the lower air level. Furthermore, the maximum and minimum levels of the square wave are always the same because the laser input power is kept constant in the operating conditions reported here. Increasing the flow rate value, moving from left to right in the upper panel of Figure 10, it is worth noting how the number of complete air–water oscillations in a time window of 15 s is essentially the same in (a) and (b), and almost doubled in (c). Further evidence is provided by the corresponding frequency value detected from the maximum peaks in the spectra. Indeed, the device is not able to make a distinction between two-phase flows at FR=0.1 mL/min and FR=0.2 mL/min, providing the same value of the fundamental frequency. Conversely, a two-phase flow moving at FR=0.3 mL/min is clearly differentiated from the others while exhibiting dynamic behaviour at an almost double carrier frequency.

**Figure 11:**
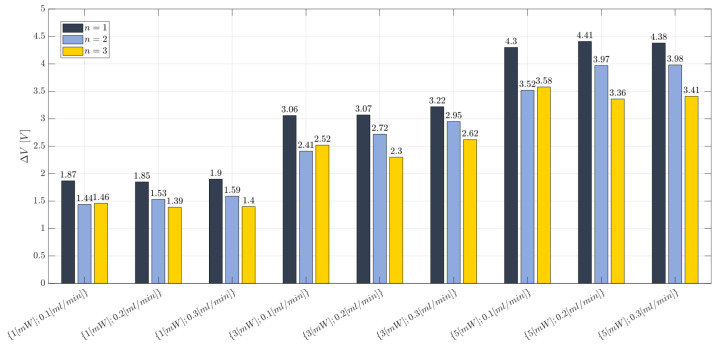
Bar plot related to the voltage difference (ΔV) measured for the BIO device at each investigated working condition (see Table 3) and replication (n=1,2,3). No error bars are reported because they are narrower than the bar height.

Moving on, the observations regarding the ΔV response are reported in the bar plot of Figure 11, for all the investigated operating conditions and replications (n=1,2,3), while, the individual value plot related to the collected observations of the voltage difference (ΔV) for the BIO device at each investigated working condition and replication (n=1,2,3) is reported in Figure S1 in the Supplementary Materials. A high repeatability for the collected responses was achieved. Thus, the developed device is suitable for the purpose and this is due to the quite steady refractive index value, i.e., a uniform cross-linked network, assessed throughout different zones of the device (see Section 3.2), with the selected manufacturing process. The ANOVA table for the ΔV response is reported in Table 6. The obtained results reveal that the laser input power is the only influential factor (factor A), (*p*-value < 0.0001). Moreover, most of the variability in the collected observations is justified by the variation in the laser input power among the different investigated levels because both the R-squared and the adjusted R-squared values are very high ($R^{2}=0.827$; $R_{adj}^{2}=0.9741$). No anomalies for the residuals from the model adequacy checking were found.

**Figure 12:**
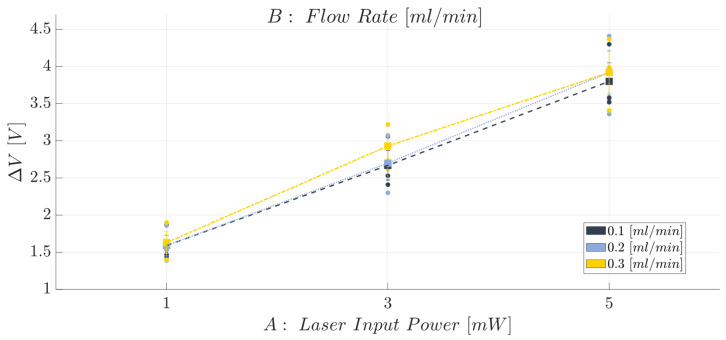
Effects diagram for the range (ΔV): BIO device.

The effects diagram for the ΔV (see Figure 12) shows that by raising the laser input power (factor A), it is possible to better discriminate between the lower air level and the higher water level: in fact, the higher the laser input power, the higher the acquired ΔV value. Thus, moving from P=1 mW up to P=3 mW, an increase of 68% was found for the ΔV, while a rise of 44% was recorded by switching from P=3 mW up to P=5 mW. The latter result is useful for suggesting the proposed device to be used even with fluids that have very similar refractive index values due to the higher ability of discrimination with a higher power value. Conversely, in line with the ANOVA results for the ΔV response, no variations were identified when switching the flow rate among the three selected levels and maintaining the factor A at a certain fixed value.

**Figure 13:**
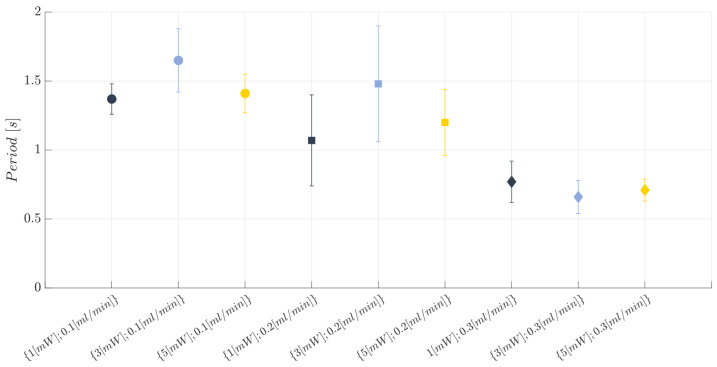
Scatter plot of the mean period (*T*) related to a complete air–water two-phase flow passage at each investigated working condition (see Table 3). The error bars represent the standard error.

**Figure 14:**
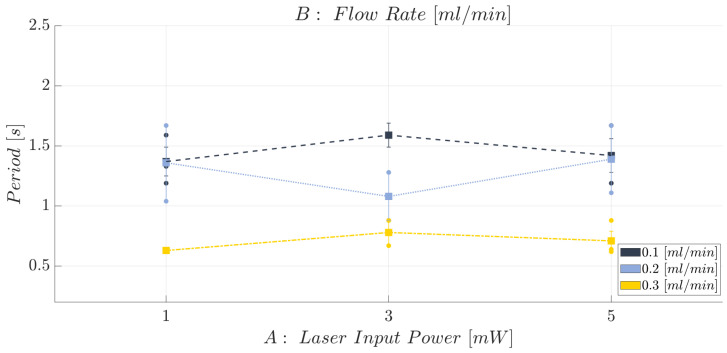
Effects diagram for the mean period associated to a complete air–water two-phase flow passage (*T*): BIO device.

The trend for the observations collected for the second investigated response, i.e., the period *T*, is shown in Figure 13, while the individual value plot related to the mean period (*T*) related to the collected observations of the complete air–water two-phase flow passage for the BIO device at each investigated working condition and replication (n=1,2,3) is reported in Figure S2 in the Supplementary Materials. The results for the ANOVA study are expressed in Table 7. According to this table, the flow rate is the only influential factor on the investigated response *T* (*p*-value < 0.05). This result is consistent with the effects diagram for *T* (see Figure 14), where no significant differences for the parameter were highlighted by varying the factor A, i.e., the laser input power. Moreover, no significant differences were found by increasing the flow rate value from 0.1 mL/min to 0.2 mL/min, thus finding that the two-phase flow has a similar hydrodynamic behaviour at these two process configurations. Conversely, a significant decrease (about 50%) was recorded by switching from 0.2 mL/min up to 0.3 mL/min. This trend is clearly visible in the scatter plot represented in Figure 13. Next, the values achieved for the R-squared and the adjusted R-squared ($R^{2}=0.7694$; $R_{adj}^{2}=0.6017$) prove that part of the variability for the collected measures is due to the variation in factor B since it is the only one influence on the considered response. From the model adequacy checking, it can be assessed that there are no anomalies for the residuals.

#### 3.2.2. HTL and BIO Resins

**Figure 15:**
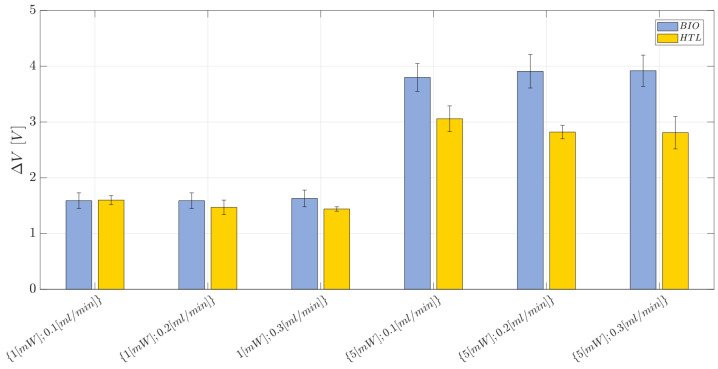
Bar plot related to the mean voltage difference (ΔV) measured for the BIO and HTL devices at each investigated working condition (see Table 4). The error bars represent the standard error.

In this section, the results obtained from the comparison analysis between the BIO and HTL devices are presented with the aim to prove that the former one is functional for the purpose, being affected by a low repeatability error. The results obtained for the response ΔV are reported in Figure 15, while the individual value plot related to the observations collected for the voltage difference (ΔV) for the BIO and HTL devices at each investigated working condition and replication (n=1,2,3) is reported in Figure S3 in the Supplementary Materials. Similar ΔV values (in terms of the average and standard error) are obtained for the BIO and HTL devices when a laser input power equal to P=1 mW is set and regardless of the flow rate considered. Conversely, when the laser input power is set at the highest level, i.e., at P=5 mW, the response ΔV is always higher for the BIO device compared to the HTL one. Thus, the BIO device better discriminates air and water providing a higher gap between the two investigated fluids, resulting in the higher values of ΔV. This result is consistent with the BIO resin’s better discrimination capability, supported by its lower estimated refractive index when compared to the HTL one [72].

These findings are also consistent with the ANOVA results, reported in Table 8, since both the laser input power (factor A) and the material (factor C) influence the response ΔV (*p*-value < 0.0001). With values of $R^{2}=0.9341$ and $R_{adj}^{2}=0.9012$, most of the variability for the acquired observations is due to the laser input power and material factors. Moreover, the model adequacy checking did not show any anomaly for the residuals.

**Figure 16:**
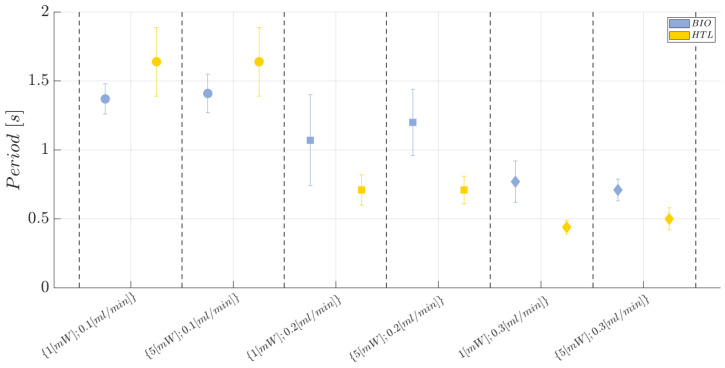
Scatter plot of the mean period (*T*) related to a complete air–water two-phase flow passage at each investigated working condition (see Table 4) for the BIO and HTL devices. The error bars represent the standard error.

Moving on to the second investigated response, that is the period (*T*), the scatter plot showing the trend is reported in Figure 16, while the individual value plot related to the mean period (*T*) related to the collected observations of the complete air–water two-phase flow passage for the BIO and HTL devices at each investigated working condition and replication (n=1,2,3) is reported in Figure S4 in the Supplementary Materials. Here it is possible to appreciate how the HTL device is more suitable for analyzing two-phase flow processes carried out at higher flow rate conditions since the acquired observations are affected by a lower repeatability error at FR = 0.2 mL/min and FR=0.3 mL/min, while a greater error bar affects the measurements carried out at the lowest level of flow rate investigated, i.e., FR=0.1 mL/min. Conversely, the BIO device is able to achieve a good hydrodynamic stability even at the lowest flow rate level tested, i.e., FR=0.1 mL/min, since the measurements are affected by a lower standard error.

**Figure 17:**
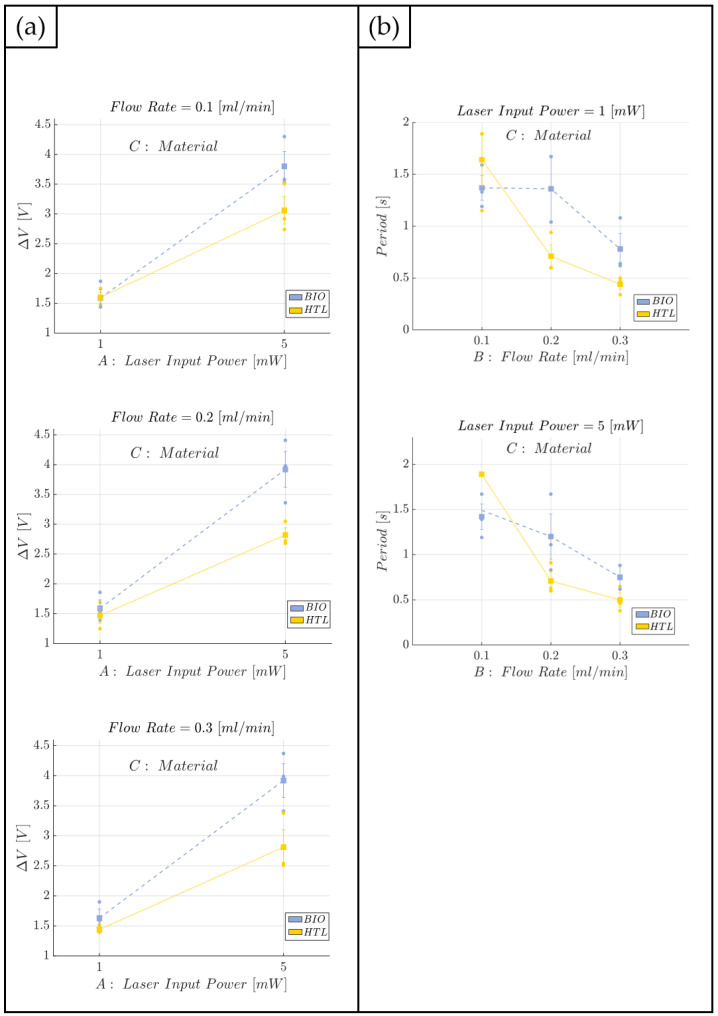
Effects diagram for the range (ΔV) (**a**) and for the mean period associated to a complete air–water two-phase flow passage (*T*) (**b**): BIO and HTL devices.

The trend described so far for the ΔV response is also confirmed and clearly shown in the effects diagram in Figure 17a, where the investigated response has the same behaviour for the two materials at 1 mW of power, while it is increased by about 34% by using the BIO device rather than the HTL one. This result is justified by the significance of the interaction AC (see ANOVA results in Table 8). Thus, the BIO device has a higher capability of discrimination, which is extremely useful when fluids with similar refractive index values are used to create the two-phase flow. In this sense, considering the biocompatible resin (BIO) is advantageous to achieve an improvement in the detection capabilities of the developed device. While, focusing on the effects diagrams reported in Figure 17b, the flow velocity is slightly higher (by about 20%) in the BIO microchannel rather than in the HTL one. This is certainly due to the stronger hydrophilic behaviour of the latter resin since its water contact angle $64.36\pm 1.63^{\circ }$ is lower than the one measured for the BIO resin, i.e., $79.07\pm 0.85^{\circ }$. The higher the hydrophilicity of the surface, the stronger the attraction of the wall toward water because water molecules interact better with bear electric charges or polar groups, which are characteristic of hydrophilic materials [73,74]. Thus, the fluid is subjected to a stronger sticky effect performed by the microchannel’s walls of the HTL device, which requires a higher flow rate to contrast any friction factor and make the hydrodynamic process stable and robust. Furthermore, focusing on the BIO device, a higher dispersion for the collected observations was found for FR=0.2 mL/min, thus proving a modest hydrodynamic variability within the microchannel, which is attributable to the slightly higher surface roughness measured for the BIO resin than the HTL one (43.56±2.62 vs. 32.32±3.25 nm). However, it is noteworthy that neither the HTL nor the BIO resins’ roughness are high enough to trigger hydrodynamic instability associated with an increase in the friction factors [78,79]. In any case, the issue of high dispersion is contrasted by raising the flow rate value at 0.3 mL/min, in the both the HTL and BIO devices. Thus, in conclusion, the HTL device achieves the best performance for FR∈{0.2;0.3} mL/min, while the BIO one achieves the best performance for FR=0.1 mL/min and FR=0.3 mL/min since it has no discrimination capability between FR=0.1 mL/min and FR=0.2 mL/min. The results discussed up to now are consistent with the results obtained from the ANOVA analysis, see Table 9. Hence, just factor B, i.e., the flow rate, is an influential factor (*p*-value <0.0001) on the mean period related to a complete two-phase flow passage (*T*). Moreover, the variability associated with the acquired responses is related to the last parameter since both $R^{2}=0.8500$ and $R_{adj}^{2}=0.7632$ are very high. Even in this case, no anomalies were found for the residuals from the model adequacy checking.

### 3.3. Cell Concentration Monitoring

**Figure 18:**
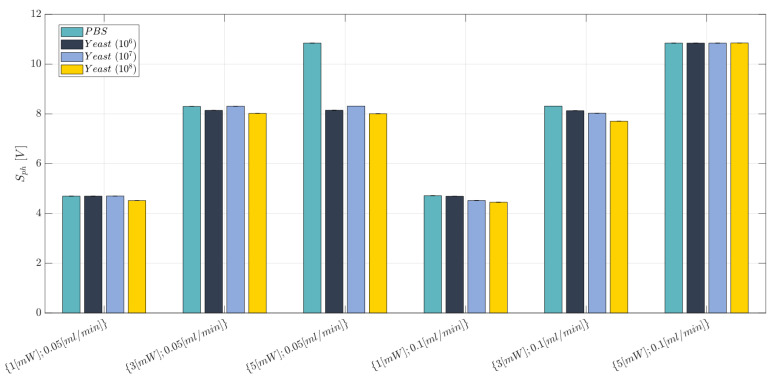
Bar plot for the $S_{ph}$ acquired for the BIO device at each investigated working condition (see Table 5). The error bars represent the standard error.

This section presents the main results obtained for the cell concentration monitoring. Here, the cell–light interaction was exploited to link the optical responses to the different cell concentrations contained in a fluid. The values associated with the acquired voltage levels $S_{ph}$ for the BIO device are reported in Figure 18 for each investigated scenario, while the individual value plot related to the mean voltage value ($S_{ph}$) of the collected observations for the BIO device under each investigated working condition and replication (n=1,2,3) is reported in Figure S5 in the Supplementary Materials. This preliminary phase was crucial to identify the optimal operative condition to carry out the detection analysis in accordance with our established method (see Section 2.2). Indeed, when the flow rate is set at its lowest level, i.e., FR=0.05 mL/min, the acquired optical signals ($S_{ph}$) show no consistent correlation between the voltage level and the investigated cell concentration. This is justified by the cells’ low velocities reached with the set input flow rate, which results in cells settling at the bottom of the channel. On the other hand, when the flow rate is set at the highest value, i.e., FR=0.1 mL/min, a decreasing trend of the acquired voltage signal related to the rising cell concentration is observed for the laser input powers equal to P=1 mW and P=3 mW. Conversely, equal values of the signal are detected from the photodiode’s acquisitions for P=5 mW when the cell concentration is varied among the b=4 considered levels. This outcome is due to the full-scale limit value of the detection instrument. Indeed, when the laser input power is set at the highest level (P=5 mW), the photodiode saturates all the acquired optical signals to a value equal to ∼10 V.

Taking into account the results obtained so far, it is reasonably legitimate to assume that a good test configuration for the MoF device developed for cell concentration monitoring should consider P∈{1,3} mW and FR=0.1 mL/min.

Once the optimal working conditions were identified, a new general replicated factorial design was investigated. Here, we consider the following design factors (independent variables):

- *Laser Input Power (factor A)*—Quantitative factor varied at two levels (a = 2) corresponding to {1, 3} mW;
- *Concentration of Yeast cells (factor B)*—Quantitative factor varied at four levels (b = 4) corresponding to {0, 10^6^, 10^7^, 10^8^} in 10 mL PBS.

**Figure 19:**
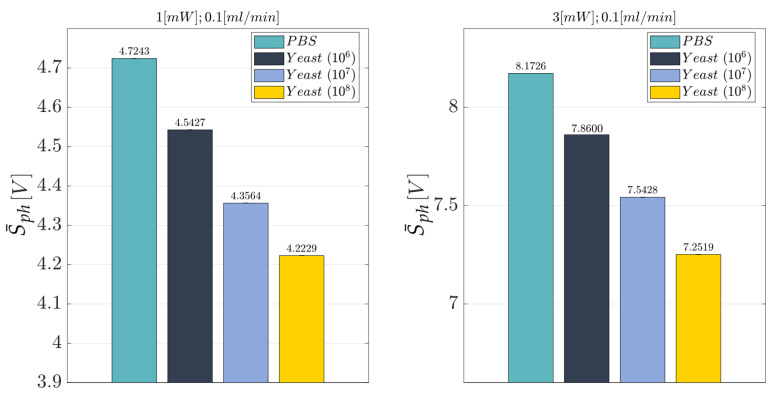
Bar plot for the overall average ${\bar{S}}_{ph}$ acquired for the BIO device at the optimal working conditions identified for the flow rate (FR=0.1 mL/min) and the laser input power (P∈{1,3} mW) (see Table 10). The average value was calculated by considering n=3 replications.

**Figure 20:**
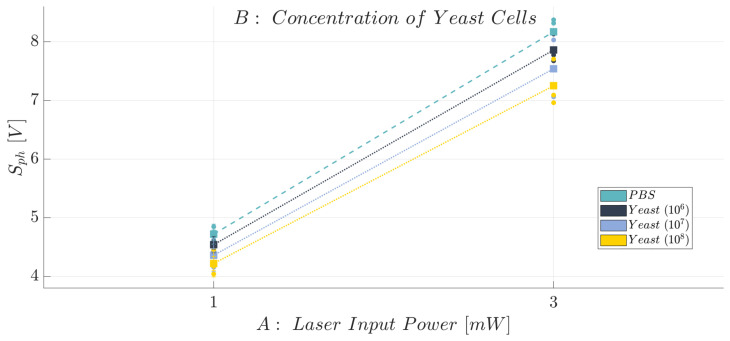
Effects diagram for the acquired optical signal ($S_{ph}$): BIO device.

The number of replications was set at n = 3, for a total of N = a × b × n = 24 experimental runs. The experimental runs were not completely randomized, but the experiments were divided into three different blocks, each of them corresponding to a different replication (n=1,2,3). The division by blocking should balance out the effect of the replications with the aim to eliminate its influence on the analysis. The experimental plan is reported in Table 10. The investigated response is the average voltage value ($S_{ph}$). The obtained results are summarized in Table 11 and reported in the bar plot in Figure 19 at the optimal working conditions identified for the flow rate (FR=0.1 mL/min) and the laser input power (P∈{1,3} mW), while the individual value plot related to the mean voltage value ($S_{ph}$) of the collected observations for the BIO device under the identified optimal working conditions and replications (n=1,2,3) is reported in Figure S6 in the Supplementary Materials. The optical voltage response shows a clear trend: the higher the cell concentration contained within the fluid (PBS), the lower the acquired optical signal intensity (${\bar{S}}_{ph}$). Further evidence of this finding is represented in the effects diagram for the $S_{ph}$ (see Figure 20). Even though the diagram shows how the optical response of the device is similar for the b=4 considered levels of the factor B, it must be highlighted that the degree of uncertainty associated with each measure is extremely low, thus proving the quite strong discrimination ability of the proposed MoF device for cell concentration monitoring. To support this, a clear behaviour of the investigated response can be inferred by looking at the four almost parallel lines that have a regular increasing trend by decreasing the concentration of yeast cells contained in the fluid (factor B) (see Figure 20). Indeed, an average decrease of about 0.17 V was found for the $S_{ph}$ by switching from 0 up to 10^6^, 10^7^, and 10^8^ cells in 10 mL of PBS fluid at a laser input power P=1 mW. Following a similar trend, an average reduction of 0.30 V was recorded by reducing the concentration of yeast cells when P=3 mW, thus confirming that the latter configuration for the power is the best one to achieve the better discrimination capability. The described trend is also confirmed from the ANOVA results (see Table 12). Both the laser input power (factor A) and the concentration of yeast cells (factor B) are influential factors (*p*-value <0.005). Most of the variability associated with the observations is related to the variation in each influential factor because both $R^{2}=0.9874$ and $R_{adj}^{2}=0.9811$ are really high. Finally, no anomalies for the residuals were identified from the model adequacy checking.

## 4. Conclusions

In this work, a 3D printed biocompatible micro-optofluidic (MoF) device was manufactured in a one-step process by using an approach based on Projection Microstereolithography (PμSL), which was previously consolidated by the authors [66].

Firstly, the MoF device was tested for the for the detection of a two-phase flow formed by two immiscible fluids, i.e., *air* and *water*. Its performances were compared to the ones of a previously developed device made of a nonbiocompatible resin (HTL) but having the same design [66]. The comparison analysis revealed that the two MoF devices have similar performances and are affected by very low repeatability error. The optimal operating conditions were determined by characterizing the materials’ chemical properties (hydrophilic behaviour and surface roughness) and by running several experimental designs to determine the effect of each process parameter (flow rate and laser input power) on the selected responses, i.e, the voltage difference ΔV and the mean period *T* associated with a complete air–water flow passage. The use of PμSL as the manufacturing technology allowed a very high accuracy of the MoF microchannel to be achieved, as confirmed by the quality monitoring analysis with a measured accuracy specification of 7.01±0.56μm, consistent with the value declared by the BMF company (10μm) for the 3D printing machine used (microArch^®^S140, BMF, Maynard, MA, USA). This permits the avoidance of hydrodynamic instability phenomena during the investigated processes.

Secondly, the 3D printed biocompatible MoF device was tested for a different kind of two-phase flow, which is the cell concentration monitoring. For this scope, an optical detection system relying on the use of a photodiode for the optical signal’s acquisition was exploited. The operating conditions used for the purpose were optimized by using proper statistical tools. The optimal values of the flow rate FR=0.1mL/min and the laser input power P∈{1,3} mW were determined to improve the discrimination between biological fluids with different concentrations of suspended cells. Since the proposed MoF device integrates both microfluidic and micro-optical components, in this work, an optical noninvasive technique suitable for cell concentration monitoring with a robust working ability, $R^{2}=0.9874$ and $R_{adj}^{2}=0.9811$, was developed and validated.

Future research activities should focus on the implementation of a suitable regression model to obtain an indirect estimation of the cell concentration level in biological fluids by measuring the voltage optical response. This simple and time-effective approach, with an almost zero computational time, will pave the way for the total-on-chip real-time analysis in biological and biomedical fields. Furthermore, several transparent 3D printable photocurable resins with a lower refractive index value should be investigated with the aim of enhancing the performance of the MoF device when optical measurements are carried out. Finally, further investigation of the use of 3D printing multimaterial approaches should be considered to fabricate totally integrated optical components within the MoF device, by following approaches used in [38,80,81]. The objective is to increase the device’s reliability by reducing the variability in the collected measurements originated by alignment issues related to the operator-dependent optical fibre insertion. 

## Figures and Tables

**Table 1:** Working principle, advantages, and drawbacks of cell concentration monitoring techniques.

Cell Concentration Monitoring Technique	Working Principle	Advantages	Drawbacks	References
**Flow** **Cytometry**	It works by suspending cells in a flowing fluid, passing them through a focused laser beam, and detecting the emitted fluorescence. By measuring the intensity and properties of this fluorescence, it quantifies cell concentration and can differentiate between different cell types based on labeled markers.	(i). High Throughput; (ii). Single-Cell Analysis; (iii). Real-Time Monitoring; (iv). Automation and Precision; (v). Labeling Flexibility	(i). Complex Instrumentation; (ii). Time-consuming sample preparation; (iii). Limited Detection Range; (iv). Complexity of Data Analysis; (v). Invasive technique (label-based using dyes).	[19,20]
**Impedance** **Spectroscopy**	It relies on measuring the electrical impedance of a microchannel or electrode when cells flow through it. As cells pass through the channel, they alter the impedance due to their size, shape, and dielectric properties. Cell concentration can be monitored by analyzing the impedance changes at different frequencies.	(i). Real-Time monitoring; (ii). Label-free; (iii). High sensitivity and accuracy; (iv). Miniaturization and integration; (v). Multiparametric analysis.	(i). High instrumentation costs; (ii). Invasive; (iii). Complex data interpretation; (iv). Sensitivity to Environmental Factors; (v). Limited cell types compatibility.	[21,23]
**Digital** **Microfluidics**	It uses a grid of electronically actuated electrodes to manipulate discrete microdroplets containing cells. By precisely moving, splitting, or merging these droplets, it enables dynamic control of cell concentrations within the droplets.	(i). Real-Time Monitoring; (ii). High Throughput; (iii). Precise Control; (iv). Integration with Sensors; (v). Reduced Sample Volume.	(i). Complex Instrumentation; (ii). Limited Droplet Size Range; (iii). Limited Sample Volume; (iv). Electrode Wear; (v). Sensitivity to Environmental Factors.	[28,29]
**Acoustic-based** **Microfluidics**	It operates by generating acoustic waves within a microchannel, so that as cells flow through it, they experience acoustic forces that push them towards specific positions or nodes within the channel. By monitoring the distribution of cells at these nodes, the method can determine cell concentration.	(i). Label-free; (ii). Noninvasive; (iii) High Precision; (iv). Cell types compatibility; (v). Real-Time monitoring.	(i). Limited Information; (ii). Complex and Expensive Equipment; (iii) Sensitivity to Environmental Factors; (iv). Limited Sample Throughput; (v). Acoustic Noise.	[30,31]
**Microscopy and** **Image Analysis**	It involves capturing images of cells within microchannels. Image analysis software then processes these images to count and analyze the cells, determining their concentration by measuring cell density or counting individual cells.	(i). Real-Time Monitoring; (ii). Noninvasive; (iii). High Precision; (iv). Multiparametric Analysis; (v). Cell types compatibility.	(i). Limited Throughput; (ii). Data Processing; (iii). High Instrumentation costs; (iv). Complexity; (v). Setup Compatibility.	[32,33]
**Optical** **Detection**	It involves illuminating cells within a microchannel with light and measuring the resulting optical signals. As cells pass through the detection zone, changes in light absorption, scattering, or fluorescence are detected and analyzed. The magnitude of these optical signals is proportional to the cell concentration, enabling quantitative monitoring and analysis.	(i). High Sensitivity; (ii). Real-Time Monitoring; (iii). Label-free; (iv). Noninvasive; (v). Integration.	(i). Sensitivity to Sample Properties; (ii). Phototoxicity; (iii). Background Noise; (iv). Temperature Sensitivity; (v). Calibration Challenges.	[34,35,36,37]

**Table 2:** Three-dimensional printing setting, washing, and post-processing procedure for the MoF device manufacturing.

3D Printing Process		
**Parameter**	**Value**	**Unit**
*Layer Thickness*	15	(μm)
*Exposure Time*	1	(s)
*Print Time*	48	(h)
**Washing**		
**Description**		
Washing in isopropyl alcohol (IPA) solution for about 5min, by changing the solutions several times.		
**Post-processing**		
**Curing Type**	**Description**	
*Thermal Curing*	at $150{\;}^{\circ }$C for 2 h	
*UV Curing*	gradient radiation with 100% UV light power (80 mW/cm^2^) for 150 s	

**Table 3:** Experimental plan for BIO device characterization: factors and levels.

Factor	Symbol	Type	Unit	Levels	Low Level (−1)	Central Level (0)	High Level (+1)
Laser Input Power	A	Quantitative	(mW)	a = 3	1	3	5
Flow Rate	B	Quantitative	(mL/min)	b = 3	0.1	0.2	0.3

**Table 4:** Experimental plan for BIO vs. HTL devices’ comparative characterization: factors and levels.

Factor	Symbol	Type	Unit	Levels	Low Level (−1)	Central Level (0)	High Level (+1)
Laser Input Power	A	Quantitative	(mW)	a = 2	1	-	5
Flow Rate	B	Quantitative	(mL/min)	b = 3	0.1	0.2	0.3
Material	C	Categorical	(-)	c = 2	HTL	-	BIO

**Table 5:** First experimental plan for cell concentration monitoring: factors and levels.

Factor	Symbol	Type	Unit	Levels	Level (1)	Level (2)	Level (3)	Level (4)
Laser Input Power	A	Quantitative	(mW)	a = 3	1	3	5	-
Concentration of Yeast Cells	B	Quantitative	(-)	b = 4	0 in 10 mL PBS	$10^{6}$ in 10 mL PBS	$10^{7}$ in 10 mL PBS	$10^{8}$ in 10 mL PBS
Flow Rate	C	Quantitative	(mL/min)	c = 2	0.05	0.1	-	-

**Table 6:** BIO device characterization: ANOVA table for the response ΔV.

Source	Sum of Squares	df	Mean Square	F-Value	*p*-Value Prob > F	
**Block**	2.07	2	1.03			
**Model**	23.43	8	2.93	113.76	<0.0001	*significant*
**(A) Laser Input Power**	23.28	2	11.64	452.05	<0.0001	
**(B) Flow Rate**	0.091	2	0.045	1.76	0.2030	
**AB**	0.062	4	0.016	0.60	0.6662	
**Residual**	0.41	16	0.026			
**Cor Total**	25.91	26				
**Std. Dev.**	0.16	**R-Squared**	0.9827			
**Mean**	2.75	**Adj R-Squared**	0.9741			
**C.V. %**	5.84	**Adeq Precision**	29.257			
**PRESS**	1.17					

**Table 7:** BIO device characterization: ANOVA table for the response *T*.

Source	Sum of Squares	df	Mean Square	F-Value	*p*-Value Prob > F	
**Block**	0.36	2	0.18			
**Model**	2.58	8	0.32	4.59	0.0113	*significant*
**(A) Laser Input Power**	0.029	2	0.015	0.21	0.8149	
**(B) Flow Rate**	2.27	2	1.14	16.17	0.00005	
**AB**	0.27	4	0.067	0.95	0.4728	
**Residual**	0.77	11	0.070			
**Cor Total**	3.72	21				
**Std. Dev.**	0.27	**R-Squared**	0.7694			
**Mean**	1.17	**Adj R-Squared**	0.6017			
**C.V. %**	22.69	**Adeq Precision**	6.856			
**PRESS**	3.58					

**Table 8:** BIO vs. HTL devices: ANOVA table for the response ΔV.

Source	Sum of Squares	df	Mean Square	F-Value	*p*-Value Prob > F	
**Block**	0.35	2	0.17			
**Model**	34.81	11	3.16	28.35	<0.0001	*significant*
**(A) Laser Input Power**	30.20	1	30.20	270.58	<0.0001	
**(B) Flow Rate**	0.032	2	0.016	0.14	0.8684	
**(C) Material**	2.65	1	2.65	23.76	<0.0001	
**AB**	5.00 × $10^{-5}$	2	2.50 × $10^{-5}$	2.24 × $10^{-4}$	0.9998	
**AC**	1.76	1	1.76	15.81	0.0006	
**BC**	0.14	**2**	0.071	0.64	0.5393	
**ABC**	0.021	**2**	0.010	0.092	0.9122	
**Residual**	2.46	**22**	0.11			
**Cor Total**	37.61	**35**				
**Std. Dev.**	0.33	**R-Squared**	0.9341			
**Mean**	2.47	**Adj R-Squared**	0.9012			
**C.V. %**	13.52	**Adeq Precision**	12.957			
**PRESS**	6.57					

**Table 9:** BIO vs. HTL devices: ANOVA table for the response *T*.

Source	Sum of Squares	df	Mean Square	F-Value	*p*-Value Prob > F	
**Block**	0.65	2	0.33			
**Model**	6.27	11	0.57	9.79	<0.0001	*significant*
**(A) Laser Input Power**	2.77 × $10^{-3}$	1	2.77 × $10^{-3}$	0.047	0.8298	
**(B) Flow Rate**	4.55	2	2.27	39.03	<0.0001	
**(C) Material**	0.27	1	0.27	4.71	0.0429	
**AB**	0.052	2	0.026	0.45	0.6446	
**AC**	0.03	1	0.03	0.52	0.4806	
**BC**	1.18	2	0.59	10.10	0.0010	
**ABC**	5.04 × $10^{-3}$	2	2.52 × $10^{-3}$	0.043	0.9578	
**Residual**	1.11	19	0.058			
**Cor Total**	8.03	32				
**Std. Dev.**	0.24	**R-Squared**	0.8500			
**Mean**	1.04	**Adj R-Squared**	0.7632			
**C.V. %**	23.21	**Adeq Precision**	10.289			
**PRESS**	3.19					

**Table 10:** Second experimental plan for cell concentration monitoring: factors and levels.

Factor	Symbol	Type	Unit	Levels	Level (1)	Level (2)	Level (3)	Level (4)
Laser Input Power	A	Quantitative	(mW)	a = 2	1	3	-	-
Concentration of Yeast Cells	B	Quantitative	(-)	b = 4	0 in 10 mL PBS	$10^{6}$ in 10 mL PBS	$10^{7}$ in 10 mL PBS	$10^{8}$ in 10 mL PBS

**Table 11:** Overall average voltage value (${\bar{S}}_{ph}$) responses measured for the BIO device at each investigated scenario (see Table 10). Measures are expressed as mean±st.err calculated on n=3 replications.

		Concentration of Yeast Cells			
		0 in 10 mL PBS	10^6^ in 10 mL PBS	10^7^ in 10 mL PBS	10^8^ in 10 mL PBS
**Laser Input** **Power (mW)**	**1**	4.7243±0.0676	4.5427±0.0787	4.3564±0.1074	4.2229±0.1217
	**3**	8.1726±0.1732	7.8600±0.1385	7.5428±0.2798	7.2519±0.2295

**Table 12:** ANOVA table for the response $S_{ph}$.

Source	Sum of Squares	df	Mean Square	F-Value	*p*-Value Prob > F	
**Block**	0.47	2	0.24			
**Model**	65.04	7	9.29	156.58	<0.0001	*significant*
**(A) Laser Input Power**	63.19	1	63.19	1064.80	<0.0001	
**(B) Concentration** **of Yeast Cells**	1.71	3	0.57	9.60	0.0011	
**AB**	0.14	3	0.048	0.81	0.5070	
**Residual**	0.83	16	0.059			
**Cor Total**	66.35	23				
**Std. Dev.**	0.24	**R-Squared**	0.9874			
**Mean**	6.08	**Adj R-Squared**	0.9811			
**C.V. %**	4.00	**Adeq Precision**	27.119			
**PRESS**	2.44					

## Data Availability

Data will be made available on request.

## Supplementary Materials

The following supporting information can be downloaded at: https://www.mdpi.com/article/10.3390/polym15224461/s1, Figure S1: Individual value plot related to the voltage difference (ΔV) collected observations for the BIO device at each investigated working condition (see Table 3) and replication (n=1,2,3); Figure S2: Individual value plot related to the mean period (*T*) related to a complete air-water two-phase flow passage collected observations for the BIO device at each investigated working condition (see Table 3) and replication (n=1,2,3); Figure S3: Individual value plot related to the voltage difference (ΔV) collected observations for the BIO and HTL devices at each investigated working condition (see Table 4) and replication (n=1,2,3); Figure S4: Individual value plot related to the mean period (*T*) related to a complete air-water two-phase flow passage collected observations for the BIO and HTL devices at each investigated working condition (see Table 4) and replication (n=1,2,3); Figure S5: Individual value plot related to the mean voltage value ($S_{ph}$) collected observations for the BIO device at each investigated working condition (see Table 5) and replication (n=1,2,3); Figure S6: Individual value plot related to the mean voltage value ($S_{ph}$) collected observations for the BIO device at each investigated working condition (see Table 10) and replication (n=1,2,3).
