# Supplementary material for: Projection Micro-Stereolithography to Manufacture a Biocompatible Micro-Optofluidic Device for Cell Concentration Monitoring
Source: Polymers (Basel). 2023 Nov 19;15(22):4461. doi: 10.3390/polym15224461 (PMC10675802; doi:10.3390/polym15224461)
Supplement: Supplementary file 1 [file polymers-15-04461-s001.zip › polymers-2690447-supplementary.pdf]

polymers

MDPI

Article

Projection Micro-Stereolithography to Manufacture a Biocompatible Micro-Optofluidic Device for Cell Concentration Monitoring

Lorena Saitta [1,*,†], Emanuela Cutuli [2,*,†], Giovanni Celano [1], Claudio Tosto [1], Dario Sanalitro [2], Francesca Guarino [3], Gianluca Cicala [1,4] and Maide Bucolo [2]

1 Department of Civil Engineering and Architecture, University of Catania, Via Santa Sofia 64, 95125 Catania, Italy; giovanni.celano@unict.it (G.C.); claudio.tosto@unict.it (C.T.); gianluca.cicala@unict.it (G.C.)
2 Department of Electrical Electronic and Computer Science Engineering, University of Catania, Via Santa Sofia 64, 95125 Catania, Italy; dario.sanalitro@unict.it (D.S); maide.bucolo@unict.it (M.B.)
3 Department of Biomedical and Biotechnological Science, University of Catania, Via Santa Sofia 89, 95123 Catania, Italy; francesca.guarino@unict.it
4 INSTM-UDR CT, Viale Andrea Doria 6, 95125 Catania, Italy
* Correspondence: lorena.saitta@phd.unict.it (L.S.); emanuela.cutuli@phd.unict.it (E.C.)
† These authors contributed equally to this work.

Keywords: 3D printing; vat photopolymerization; photocurable biocompatible resins; micro-optics; microfluidics; two-phase flow detection; cell concentration detection

check for updates

Citation: Saitta, L.; Cutuli, E.; Celano, G.; Tosto, C.; Sanalitro, D.; Guarino, F.; Cicala, G.; Bucolo, M. Projection Micro-Stereolithography to Manufacture a Biocompatible Micro-Optofluidic Device for Cell Concentration Monitoring. *Polymers* **2023**, *15*, 4461. https://doi.org/10.3390/polym15224461

Academic Editor: Qinghua Wei

Received: 14 October 2023
Revised: 15 November 2023
Accepted: 16 November 2023
Published: 19 November 2023

Copyright: © 2023 by the authors. Licensee MDPI, Basel, Switzerland. This article is an open access article distributed under the terms and conditions of the Creative Commons Attribution (CC BY) license (https://creativecommons.org/licenses/by/4.0/).

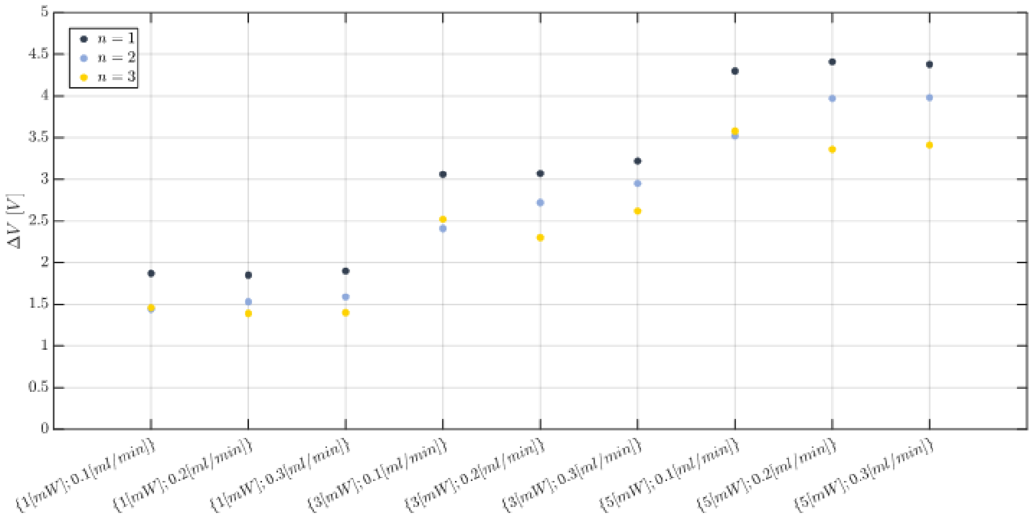

Figure S 1. Individual value plot related to the voltage difference (ΔV) collected observations for the BIO device at each investigated working condition (see Table 3) and replication ($n = 1, 2, 3$).

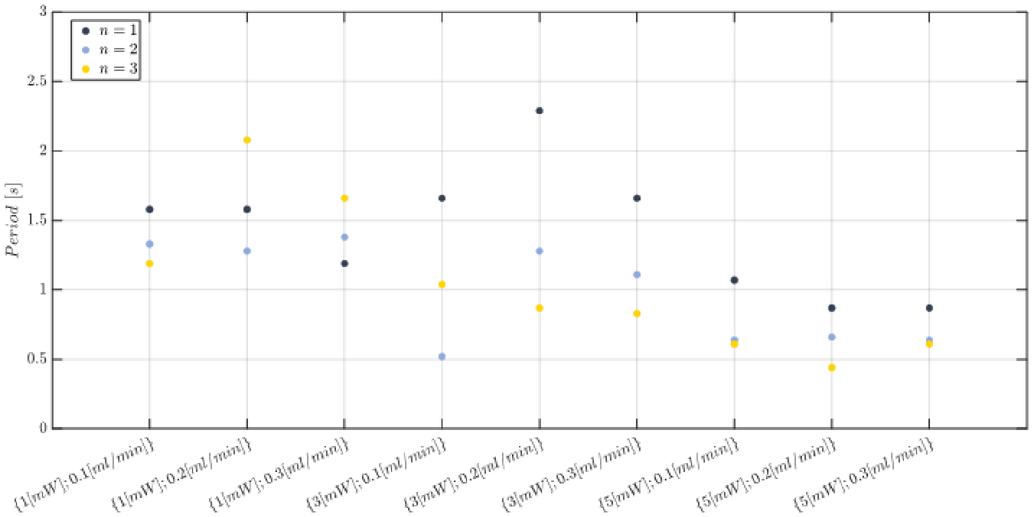

Figure S 2. Individual value plot related to the mean period (T) related to a complete air-water two-phase flow passage collected observations for the BIO device at each investigated working condition (see Table 3) and replication ($n = 1, 2, 3$).

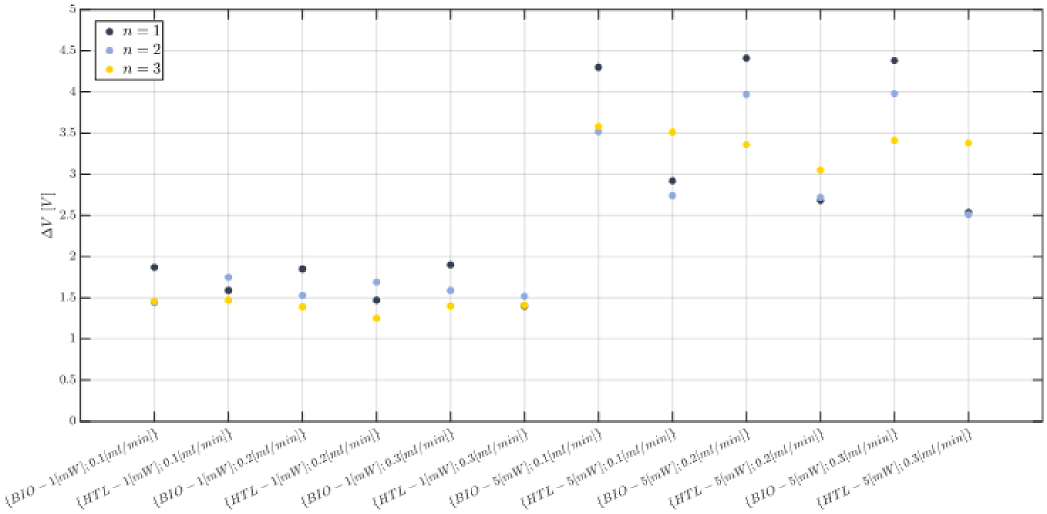

Figure S 3. Individual value plot related to the voltage difference (ΔV) collected observations for the BIO and HTL devices at each investigated working condition (see Table 4) and replication ($n = 1, 2, 3$).

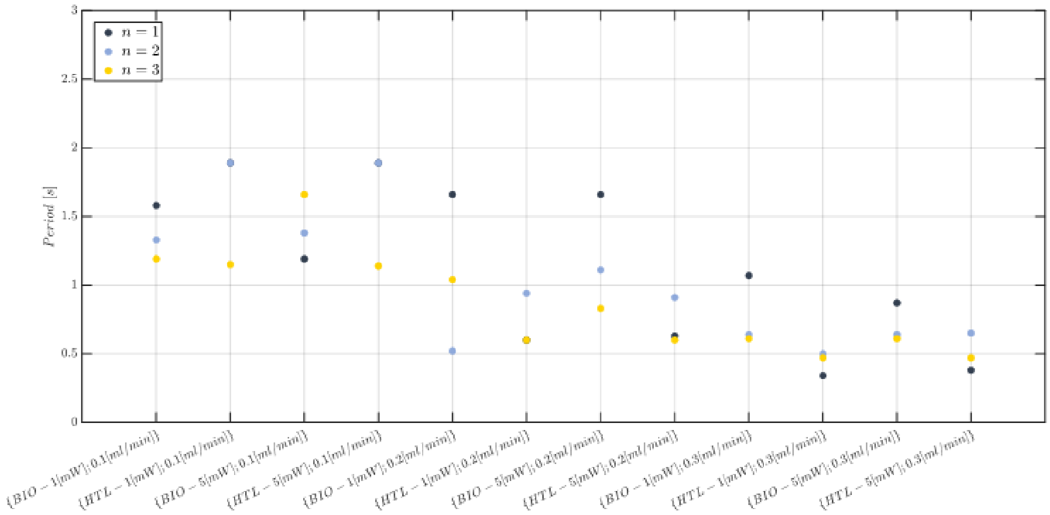

Figure S 4. Individual value plot related to the mean period (T) related to a complete air-water two-phase flow passage collected observations for the BIO and HTL devices at each investigated working condition (see Table 4) and replication ($n = 1, 2, 3$).

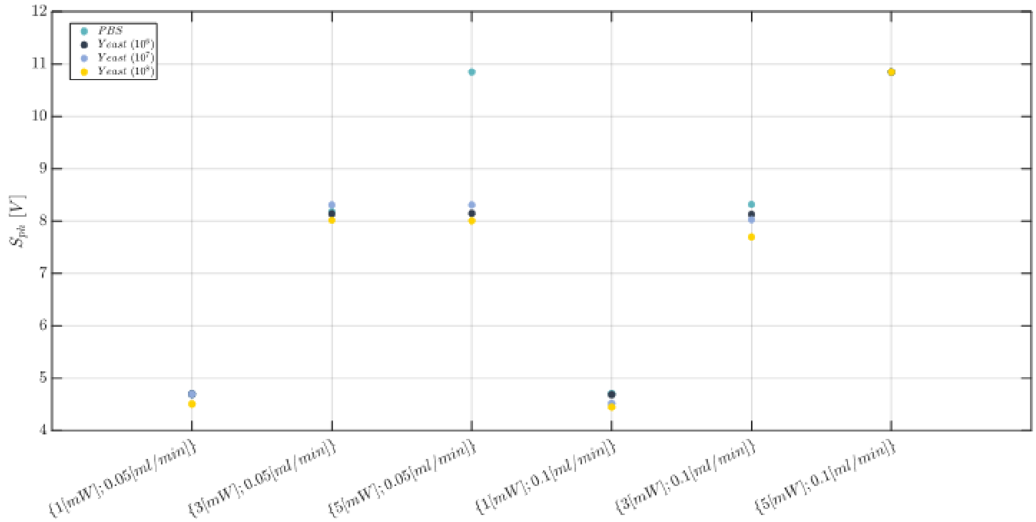

Figure S 5. Individual value plot related to the mean voltage value (S_{ph}) collected observations for the BIO device at each investigated working condition (see Table 5) and replication ($n = 1, 2, 3$).

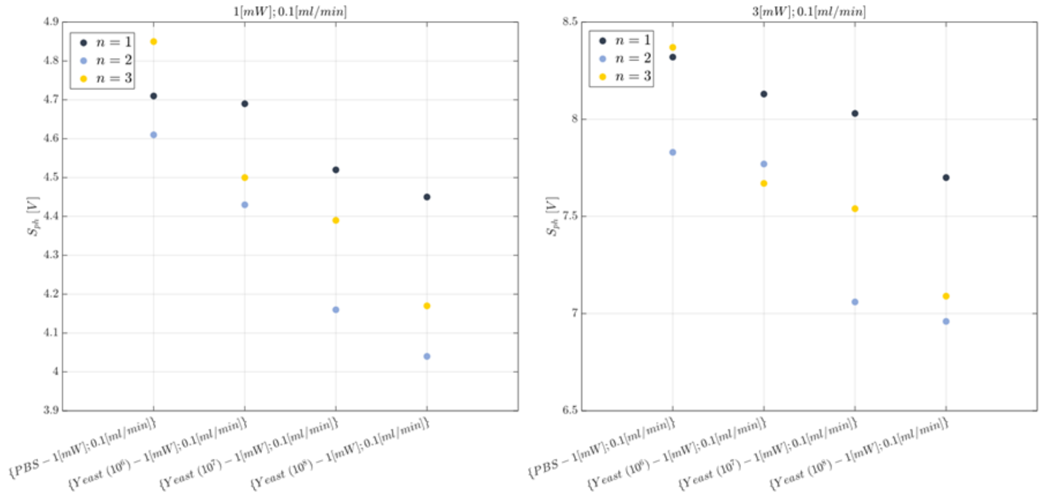

Figure S 6. Individual value plot related to the mean voltage value (S_{ph}) collected observations for the BIO device at each investigated working condition (see Table 10) and replication ($n = 1, 2, 3$).
